# Crystal structure of hexa-μ-chlorido-μ_4_-oxido-tetra­kis­{[1-(2-hy­droxy­eth­yl)-2-methyl-5-nitro-1*H*-imidazole-κ*N* ^3^]copper(II)} containing short NO_2_⋯NO_2_ contacts

**DOI:** 10.1107/S2056989019008570

**Published:** 2019-06-25

**Authors:** Ja-Shin Wu, Daniel G. Shlian, Joshua H. Palmer, Rita K. Upmacis

**Affiliations:** a Department of Chemistry & Physical Sciences, Pace University, New York, NY 10038, USA; b Department of Chemistry, Columbia University, New York, NY 10027, USA; c Dept. of Chemistry & Physical Sciences, Pace University, New York, NY 10038, USA

**Keywords:** crystal structure, tetra­nuclear copper, metronidazole, bridging chloride, NO_2_ Inter­actions

## Abstract

The title tetra­nuclear copper complex, [Cu_4_Cl_6_O(C_6_H_9_N_3_O_3_)_4_] or [Cu_4_Cl_6_O­(MET)_4_] [MET is 1-(2-hy­droxy­eth­yl)-2-methyl-5-nitro-1*H*-imidazole or metronidazole], contains a tetra­hedral arrangement of copper(II) ions. Each copper atom is also linked to the other three copper atoms in the tetra­hedron *via* bridging chloride ions. A fifth coordination position on each metal atom is occupied by a nitro­gen atom of the monodentate MET ligand. The result is a distorted CuCl_3_NO trigonal–bipyramidal coordination polyhedron with the axial positions occupied by oxygen and nitro­gen atoms. The extended structure displays O—H⋯O hydrogen bonding, as well as unusual short O⋯N inter­actions [2.775 (4) Å] between the nitro groups of adjacent clusters that are oriented perpendicular to each other. The scattering contribution of disordered water and methanol solvent mol­ecules was removed using the SQUEEZE procedure [Spek (2015 ▸). *Acta Cryst*. C**71**, 9–16] in *PLATON* [Spek (2009 ▸). *Acta Cryst.* D**65**, 148–155].

## Abstract 2

The title tetra­nuclear cluster contains a tetra­hedral arrangement of copper(II) ions bonded to a central oxygen atom. The extended structure shows short O⋯N inter­actions between the nitro groups of adjacent clusters, which are oriented perpendicular to each other in a manner that has previously been described as an O_NO2_⋯π(N)_NO2_ inter­action.

## Chemical context   

Metronidazole (C_6_H_9_N_3_O_3_; MET) is a medication that was discovered to be effective against both bacteria and parasites more than 50 years ago (Samuelson, 1999 ▸). MET is currently incorporated in the World Health Organization (WHO) list of essential medicines, *i.e.* medications that are considered to be effective and safe to meet the most important needs in a health system (WHO, 2015 ▸). Despite the widespread use of MET as a drug, relatively little structural data concerning its inter­actions with metal ions exist, and there are few structurally characterized copper compounds of MET (Galván-Tejada *et al.*, 2002 ▸; Barba-Behrens *et al.*, 1991 ▸; Athar *et al.*, 2005 ▸; Ratajczak-Sitarz *et al.*, 1998 ▸; Bharti *et al.*, 2002 ▸). Our recent work has sought to develop further metal–MET chemistry and we have reported structures containing Cu (Palmer *et al.*, 2015 ▸; Quinlivan & Upmacis, 2016 ▸), as well as Ag (Palmer & Upmacis, 2015 ▸) and Au (Quinlivan *et al.*, 2015 ▸). Tetra­nuclear copper(II) compounds of the form [Cu_4_O*X*_6_ *L*_4_] are relatively well known, with the first example described in 1996 (Bertrand & Kelley, 1966 ▸). In this regard, although the structure of a [Cu_4_O*X*_6_ *L*_4_] structure, where *L* = imidazole, has been previously described (Atria *et al.*, 1999 ▸), a counterpart containing *L* = MET has not been reported. Herein, we describe the structure of a tetra­nuclear Cu–MET complex [Cu_4_Cl_6_O(MET)_4_] that is obtained by the reaction of anhydrous copper(I) chloride with MET in MeOH under aerobic conditions.
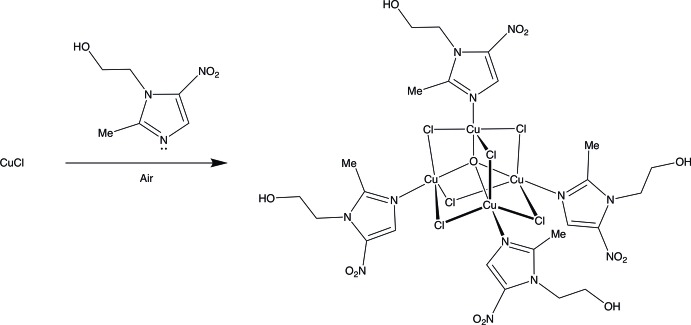


## Structural commentary   

**Figure 1:**
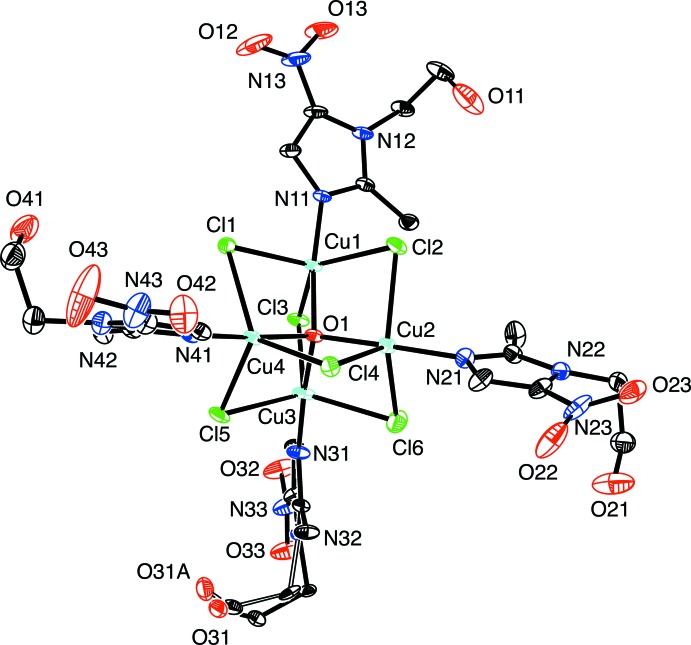
The mol­ecular structure of [Cu_4_Cl_6_O(MET)_4_]. For clarity, hydrogen atoms have been omitted. The eth­oxy group of the MET ligand attached to Cu3 (comprising C34, C35 and O31) is disordered over two sets of sites in a 0.515 (19):0.485 (19) ratio.

The structure of the [Cu_4_Cl_6_O(MET)_4_] complex is shown in Fig. 1 ▸. Four copper atoms are arranged around an oxygen atom in a tetra­hedral fashion, with Cu—O distances ranging from 1.8960 (18) to 1.913 (2) Å. The Cu—O—Cu angles range from 108.36 (10) to 110.80 (9)°, indicating a fairly uniform tetra­hedron with little distortion. In fact, the degree of distortion from a tetra­hedral arrangement can be readily quan­ti­fied by the τ_4_ four-coordinate geometry index that is reported and discussed elsewhere (Yang *et al.*, 2007 ▸; Palmer *et al.*, 2015 ▸, Brescia *et al.*, 2018 ▸). Briefly, τ_4_ is obtained from the expression, τ_4_ = [360 − (α +  β)]/141, where α and β represent the two largest angles; a τ_4_ value of 1.00 indicates an idealized tetra­hedral geometry, whereas a value of 0.00 indicates an idealized square-planar geometry. In the title complex, α = 110.80 (9)° and β = 109.55 (9)°, such that τ_4_ is 0.990, which indicates negligible deviation from a tetra­hedral geometry for oxygen (Yang *et al.*, 2007 ▸).

Each of the four copper atoms is linked to the other three copper atoms *via* three chloride bridges, with the Cu—Cl bridging distances varying from 2.3579 (10) to 2.4435 (9) Å (for Cu2—Cl6 and Cu1—Cl2, respectively). Each copper atom is also bound to a nitro­gen atom of a MET ligand. The Cu—N lengths range from 1.949 (2) to 1.972 (3) Å (for Cu1—N11 and Cu4—N41, respectively). Thus, each copper atom sits within a trigonal–bipyramidal arrangement, with the oxygen and nitro­gen atoms forming the axial coordination points, and the bridging chloride ligands occupying the equatorial plane. The trigonal–bipyramidal structure is somewhat distorted, as indicated by the fact that the O—Cu—N angles are less than 180°, ranging from 173.12 (10) to 176.91 (10)° (for O1—Cu1—N11 and O1—Cu2—N21, respectively), and the Cl—Cu—Cl angles differ significantly from 120°, ranging from 109.97 (3) to 134.02 (3)° (for Cl2—Cu2—Cl4 and Cl3—Cu1—Cl2, respectively). Furthermore, the O—Cu—Cl angles are all less than 90°, ranging from 83.33 (6) to 86.13 (6)° (for O1—Cu1—Cl2 and O1—Cu—Cl1, respectively), indicating that the equatorial chloride ligands are displaced slightly more towards the axial oxygen atom in the center of the mol­ecule, than towards the nitro­gen-containing ligand in the opposite axial position.

The τ_5_ geometry index is a general descriptor of five-coordinate mol­ecules and provides a way to determine the extent of distortion of a mol­ecule from trigonal bipyramidal to square pyramidal (Addison *et al.*, 1984 ▸). The τ_5_ geometry index is calculated by using the equation: τ_5_ = (β − α)/60, where β − α is the difference between the two largest angles (Addison *et al.*, 1984 ▸; Palmer & Parkin, 2014 ▸). The values for τ_5_ are calculated to be 0.65 (Cu1), 0.74 (Cu2), 0.84 (Cu3) and 0.73 (Cu4) for the five-coordinate copper centers, giving an average τ_5_ value of 0.74. The τ_5_ values obtained indicate that the copper-centered structures are closer to an idealized trigonal–bipyramidal (1.00) than a square-pyramidal geometry (0.00).

## Supra­molecular features   

**Figure 2:**
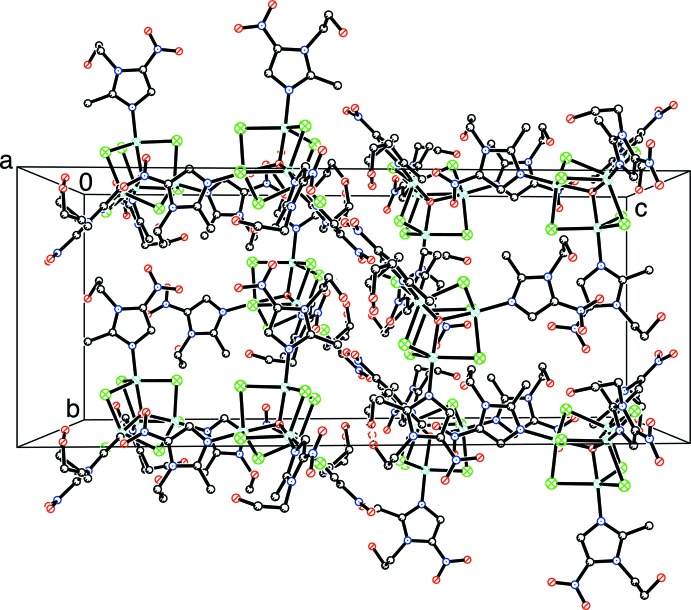
Unit-cell packing of [Cu_4_Cl_6_O(MET)_4_] viewed down [100].

**Figure 3:**
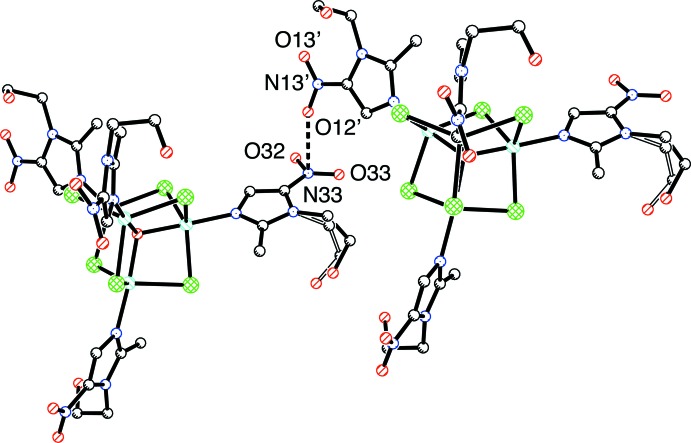
Detail of the O⋯N inter­action between the nitro groups of adjacent clusters.

Fig. 2 ▸ shows the packing in the unit cell. As well as the O—H⋯O hydrogen bonds shown in Table 1 ▸, O11—H11*A* and O21—H21*A* probably form links to the disordered solvent mol­ecules removed with SQUEEZE (see *Experimental*). The most inter­esting observation is the existence of short O⋯N inter­actions between the N13/O12/O13 and N33/O32/O33 nitro groups of adjacent clusters that are oriented perpendicular to each other, as illustrated in Fig. 3 ▸ with O12⋯N33 = 2.775 (4) Å. This type of contact has previously been described as an O_NO2_⋯π(N)_NO2_ inter­action (Daszkiewicz, 2013 ▸); such contacts are typically shorter than 3 Å.

## Database survey   

The tetra­nuclear copper motif, *L*_4_Cu_4_Cl_6_O, where *L* is a nitro­gen-containing Lewis base ligand, is common. For instance, several structures have been reported in which *L* contains either an imidazole or substituted imidazole moiety (Clegg *et al.*, 1988 ▸; Norman *et al.*, 1989 ▸ Erdonmez *et al.*, 1990 ▸; Atria *et al.*, 1999 ▸; Cortés *et al.*, 2006 ▸; Chiarella *et al.*, 2009 ▸, 2010 ▸; She *et al.*, 2010 ▸) or a benzimidazole moiety (Tosik *et al.*, 1991 ▸ Zhang *et al.*, 2003 ▸; Jian *et al.*, 2004 ▸; Li *et al.*, 2011 ▸).

The title compound [Cu_4_Cl_6_O(MET)_4_] contains Cu—*X* distances that are similar to those in [Cu_4_Cl_6_O(imidazole)_4_] (Atria *et al.*, 1999 ▸). For example, the Cu—O distances in [Cu_4_Cl_6_O(MET)_4_] are 1.8960 (18)–1.913 (2) Å, compared to 1.903 (4)–1.924 (4) Å for [Cu_4_Cl_6_O(imidazole)_4_]. Likewise, the Cu—Cl distances in [Cu_4_Cl_6_O(MET)_4_] are 2.3579 (10)–2.4435 (9) Å, compared to 2.374 (2)–2.564 (2) Å for [Cu_4_Cl_6_O(imidazole)_4_]. Moreover, the Cu—N distances in [Cu_4_Cl_6_O(MET)_4_] are 1.949 (2)–1.972 (3) Å, compared to 1.934 (6)–1.961 (6) Å.

## Synthesis and crystallization   

Anhydrous copper(I) chloride (0.015 g, 0.00015 mol) was mixed with MET (0.05075 g, 0.00030 mol) in methanol (2 ml) in a glass vial, forming a dark olive-colored solution. After allowing the solution to evaporate for eight days, gold-colored plates, suitable for X-ray diffraction, were obtained.

## Refinement   

Crystal data, data collection and structure refinement details are summarized in Table 2 ▸. Hydrogen atoms on carbon were placed in calculated positions (C—H = 0.95–1.00 Å) and included as riding contributions with isotropic displacement parameters *U*_iso_(H) = 1.2*U*_eq_(C*sp* ^2^) or 1.5*U*_eq_(C*sp* ^3^). Atoms C34, C35 and O31 and their attached H atoms were modeled as disordered over two sets of sites in a 0.515 (19):0.485 (19) ratio. The structure contains two methanol mol­ecules and one water mol­ecule, but they are disordered and were removed by the SQUEEZE procedure in *PLATON* (Spek, 2015 ▸); the stated crystal data (*M*_r_, μ, etc.) only refer to the main mol­ecule.

## Supplementary Material

Crystal structure: contains datablock(s) I. DOI: 10.1107/S2056989019008570/hb7801sup1.cif

Structure factors: contains datablock(s) I. DOI: 10.1107/S2056989019008570/hb7801Isup2.hkl

CCDC reference: 1923275

Additional supporting information:  crystallographic information; 3D view; checkCIF report

## Figures and Tables

**Table 1:** Hydrogen-bond geometry (Å, °)

*D*—H⋯*A*	*D*—H	H⋯*A*	*D*⋯*A*	*D*—H⋯*A*
O41—H41*A*⋯O31^i^	0.89 (2)	2.13 (3)	2.738 (8)	125 (2)

Symmetry code: (i) 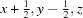.

**Table 2:** Experimental details

Crystal data	
Chemical formula	[Cu_4_Cl_6_O(C_6_H_9_N_3_O_3_)_4_]
*M*_r_	1167.51
Crystal system, space group	Monoclinic, *C*2/*c*
Temperature (K)	130
*a*, *b*, *c* (Å)	22.125 (3), 13.361 (2), 32.633 (5)
β (°)	94.752 (2)
*V* (Å^3^)	9613 (3)
*Z*	8
Radiation type	Mo *K*α
μ (mm^−1^)	2.14
Crystal size (mm)	0.36 × 0.20 × 0.10

Data collection	
Diffractometer	Bruker APEXII CCD
Absorption correction	Multi-scan (*SADABS*; Bruker, 2008 ▸)
*T*_min_, *T*_max_	0.586, 0.746
No. of measured, independent and observed [*I* > 2σ(*I*)] reflections	78050, 15003, 11100
*R*_int_	0.048
(sin θ/λ)_max_ (Å^−1^)	0.720

Refinement	
*R*[*F* ^2^ > 2σ(*F* ^2^)], *wR*(*F* ^2^), *S*	0.045, 0.118, 1.03
No. of reflections	15003
No. of parameters	579
No. of restraints	120
H-atom treatment	H atoms treated by a mixture of independent and constrained refinement
Δρ_max_, Δρ_min_ (e Å^−3^)	1.55, −1.09

Computer programs: *APEX2* and), *SAINT* (Bruker, 2008 ▸), *SHELXS97* (Sheldrick 2008 ▸), *SHELXL2014* (Sheldrick, 2015 ▸) and *SHELXTL* (Sheldrick, 2008 ▸).

## supplementary crystallographic information

### Crystal data

**Table:**

[Cu_4_Cl_6_O(C_6_H_9_N_3_O_3_)_4_]	*F*(000) = 4688
*M**_r_* = 1167.51	*D*_x_ = 1.613 Mg m^−^^3^
Monoclinic, *C*2/*c*	Mo *K*α radiation, λ = 0.71073 Å
*a* = 22.125 (3) Å	Cell parameters from 9836 reflections
*b* = 13.361 (2) Å	θ = 2.2–29.8°
*c* = 32.633 (5) Å	µ = 2.14 mm^−^^1^
β = 94.752 (2)°	*T* = 130 K
*V* = 9613 (3) Å^3^	Plate, gold
*Z* = 8	0.36 × 0.20 × 0.10 mm

### Data collection

**Table:**

Bruker APEXII CCD diffractometer	11100 reflections with *I* > 2σ(*I*)
φ and ω scans	*R*_int_ = 0.048
Absorption correction: multi-scan (SADABS; Bruker, 2008)	θ_max_ = 30.8°, θ_min_ = 1.3°
*T*_min_ = 0.586, *T*_max_ = 0.746	*h* = −31→31
78050 measured reflections	*k* = −19→19
15003 independent reflections	*l* = −46→46

### Refinement

**Table:**

Refinement on *F*^2^	Primary atom site location: structure-invariant direct methods
Least-squares matrix: full	Hydrogen site location: mixed
*R*[*F*^2^ > 2σ(*F*^2^)] = 0.045	H atoms treated by a mixture of independent and constrained refinement
*wR*(*F*^2^) = 0.118	*w* = 1/[σ^2^(*F*_o_^2^) + (0.0497*P*)^2^ + 31.4385*P*] where *P* = (*F*_o_^2^ + 2*F*_c_^2^)/3
*S* = 1.03	(Δ/σ)_max_ = 0.002
15003 reflections	Δρ_max_ = 1.55 e Å^−^^3^
579 parameters	Δρ_min_ = −1.09 e Å^−^^3^
120 restraints	

### Special details

**Table:**

Geometry. All esds (except the esd in the dihedral angle between two l.s. planes) are estimated using the full covariance matrix. The cell esds are taken into account individually in the estimation of esds in distances, angles and torsion angles; correlations between esds in cell parameters are only used when they are defined by crystal symmetry. An approximate (isotropic) treatment of cell esds is used for estimating esds involving l.s. planes.

### Fractional atomic coordinates and isotropic or equivalent isotropic displacement parameters (Å^2^)

**Table:**

	*x*	*y*	*z*	*U*_iso_*/*U*_eq_	Occ. (<1)
Cu1	0.80290 (2)	−0.01300 (3)	0.39169 (2)	0.02214 (8)	
Cu2	0.70660 (2)	−0.01915 (3)	0.31768 (2)	0.02614 (8)	
Cu3	0.66594 (2)	0.02107 (3)	0.40449 (2)	0.03036 (9)	
Cu4	0.71326 (2)	−0.19124 (3)	0.38380 (2)	0.02342 (8)	
Cl1	0.81567 (3)	−0.18214 (5)	0.41707 (2)	0.02840 (14)	
Cl2	0.81362 (3)	−0.00755 (7)	0.31780 (2)	0.03451 (17)	
Cl3	0.75591 (3)	0.09683 (6)	0.43943 (2)	0.03046 (16)	
Cl4	0.67511 (3)	−0.19355 (6)	0.31175 (2)	0.03082 (15)	
Cl5	0.63812 (3)	−0.13927 (7)	0.42920 (2)	0.03596 (17)	
Cl6	0.63258 (5)	0.09252 (9)	0.33861 (3)	0.0547 (3)	
N11	0.88549 (10)	0.03600 (18)	0.40341 (7)	0.0233 (5)	
N12	0.96498 (10)	0.13435 (19)	0.39975 (9)	0.0302 (5)	
N13	1.04472 (14)	0.0232 (3)	0.43287 (15)	0.0646 (12)	
N21	0.68926 (12)	0.0204 (2)	0.26024 (8)	0.0307 (5)	
N22	0.67466 (12)	0.1147 (2)	0.20463 (8)	0.0312 (6)	
N23	0.62700 (16)	−0.0128 (3)	0.15652 (10)	0.0497 (9)	
N31	0.60566 (11)	0.0980 (2)	0.43173 (8)	0.0316 (6)	
N32	0.51725 (10)	0.15805 (18)	0.44639 (7)	0.0243 (5)	
N33	0.55277 (14)	0.2806 (3)	0.50054 (11)	0.0513 (9)	
N41	0.70835 (10)	−0.33784 (19)	0.38980 (7)	0.0254 (5)	
N42	0.71449 (13)	−0.4914 (2)	0.41434 (9)	0.0355 (6)	
N43	0.71274 (19)	−0.5839 (3)	0.34682 (11)	0.0591 (10)	
O1	0.72212 (8)	−0.05076 (15)	0.37460 (6)	0.0222 (4)	
O11	0.9926 (2)	0.1888 (4)	0.31860 (12)	0.0965 (15)	
H11A	0.991 (3)	0.228 (4)	0.2983 (14)	0.145*	
O12	1.05456 (14)	−0.0584 (3)	0.44694 (19)	0.125 (2)	
O13	1.08424 (12)	0.0857 (3)	0.43043 (14)	0.0822 (12)	
O21	0.55815 (16)	0.1846 (3)	0.17440 (17)	0.0943 (14)	
H21A	0.5252 (8)	0.206 (4)	0.1839 (17)	0.141*	
O22	0.59231 (17)	−0.0862 (2)	0.15657 (10)	0.0714 (11)	
O23	0.64160 (13)	0.0312 (3)	0.12553 (8)	0.0606 (9)	
O32	0.59897 (13)	0.3128 (3)	0.52030 (10)	0.0717 (11)	
O33	0.50093 (13)	0.3067 (3)	0.50600 (10)	0.0683 (10)	
O41	0.81469 (16)	−0.5364 (3)	0.47601 (15)	0.0891 (14)	
H41A	0.8525 (9)	−0.5590 (19)	0.479 (2)	0.134*	
O42	0.70514 (17)	−0.5744 (2)	0.30998 (9)	0.0643 (9)	
O43	0.7238 (3)	−0.6628 (3)	0.36399 (13)	0.139 (2)	
C11	0.90440 (12)	0.1254 (2)	0.39148 (9)	0.0261 (6)	
C12	0.93477 (13)	−0.0145 (2)	0.42001 (11)	0.0333 (7)	
H12A	0.9349	−0.0801	0.4312	0.040*	
C13	0.98391 (13)	0.0457 (3)	0.41783 (12)	0.0380 (8)	
C14	1.00057 (14)	0.2241 (3)	0.39029 (12)	0.0403 (8)	
H14A	1.0302	0.2388	0.4139	0.048*	
H14B	0.9729	0.2822	0.3864	0.048*	
C15	1.03387 (19)	0.2108 (4)	0.35240 (16)	0.0627 (13)	
H15A	1.0563	0.2729	0.3469	0.075*	
H15B	1.0636	0.1557	0.3567	0.075*	
C16	0.86509 (14)	0.2053 (3)	0.37248 (12)	0.0369 (7)	
H16A	0.8229	0.1822	0.3698	0.055*	
H16B	0.8780	0.2213	0.3452	0.055*	
H16C	0.8684	0.2652	0.3899	0.055*	
C21	0.69669 (14)	0.1114 (3)	0.24465 (9)	0.0311 (6)	
C22	0.66075 (16)	−0.0364 (3)	0.22997 (11)	0.0382 (8)	
H22A	0.6493	−0.1046	0.2323	0.046*	
C23	0.65153 (15)	0.0209 (3)	0.19587 (10)	0.0355 (7)	
C24	0.66535 (15)	0.2064 (3)	0.18002 (11)	0.0398 (8)	
H24A	0.6692	0.1905	0.1507	0.048*	
H24B	0.6972	0.2557	0.1889	0.048*	
C25	0.60375 (18)	0.2517 (3)	0.18455 (15)	0.0514 (10)	
H25A	0.6014	0.2739	0.2133	0.062*	
H25B	0.5983	0.3113	0.1666	0.062*	
C26	0.7245 (2)	0.1984 (3)	0.26728 (12)	0.0515 (10)	
H26A	0.7510	0.1746	0.2908	0.077*	
H26B	0.6925	0.2407	0.2771	0.077*	
H26C	0.7483	0.2373	0.2489	0.077*	
C31	0.54558 (12)	0.0912 (2)	0.42365 (9)	0.0247 (5)	
C32	0.61649 (13)	0.1716 (2)	0.46056 (9)	0.0299 (6)	
H32A	0.6552	0.1934	0.4719	0.036*	
C33	0.56247 (14)	0.2077 (2)	0.47002 (10)	0.0308 (6)	
C34	0.4518 (4)	0.1791 (10)	0.4409 (4)	0.027 (2)	0.515 (19)
H34A	0.4346	0.1503	0.4145	0.033*	0.515 (19)
H34B	0.4451	0.2523	0.4399	0.033*	0.515 (19)
C35	0.4205 (4)	0.1352 (8)	0.4754 (3)	0.034 (2)	0.515 (19)
H35A	0.4351	0.1686	0.5014	0.041*	0.515 (19)
H35B	0.3763	0.1469	0.4706	0.041*	0.515 (19)
O31	0.4317 (4)	0.0314 (7)	0.4788 (3)	0.040 (2)	0.515 (19)
H31A	0.4316 (19)	0.012 (3)	0.5031 (8)	0.060*	0.515 (19)
C34A	0.4496 (4)	0.1552 (12)	0.4507 (5)	0.036 (3)	0.485 (19)
H34D	0.4353	0.2234	0.4568	0.044*	0.485 (19)
H34E	0.4283	0.1335	0.4243	0.044*	0.485 (19)
C35A	0.4336 (5)	0.0858 (14)	0.4841 (4)	0.053 (4)	0.485 (19)
H35D	0.4523	0.1103	0.5108	0.063*	0.485 (19)
H35E	0.3890	0.0857	0.4854	0.063*	0.485 (19)
O31A	0.4535 (6)	−0.0129 (10)	0.4775 (2)	0.054 (3)	0.485 (19)
H31D	0.483 (6)	−0.012 (11)	0.489 (4)	0.081*	0.485 (19)
C36	0.51388 (13)	0.0216 (3)	0.39382 (11)	0.0357 (7)	
H36A	0.5428	−0.0279	0.3850	0.053*	
H36B	0.4966	0.0594	0.3699	0.053*	
H36C	0.4813	−0.0126	0.4068	0.053*	
C41	0.71482 (13)	−0.3938 (2)	0.42409 (9)	0.0287 (6)	
C42	0.70446 (13)	−0.4025 (2)	0.35718 (9)	0.0281 (6)	
H42A	0.6992	−0.3840	0.3290	0.034*	
C43	0.70926 (16)	−0.4966 (2)	0.37164 (10)	0.0352 (7)	
C44	0.71595 (19)	−0.5751 (3)	0.44430 (12)	0.0480 (9)	
H44A	0.7000	−0.5519	0.4701	0.058*	
H44B	0.6897	−0.6302	0.4330	0.058*	
C45	0.7785 (2)	−0.6121 (4)	0.45304 (15)	0.0602 (11)	
H45A	0.7781	−0.6746	0.4693	0.072*	
H45B	0.7964	−0.6269	0.4269	0.072*	
C46	0.71969 (17)	−0.3551 (3)	0.46674 (10)	0.0393 (8)	
H46A	0.7583	−0.3765	0.4809	0.059*	
H46B	0.6861	−0.3813	0.4813	0.059*	
H46C	0.7179	−0.2818	0.4662	0.059*	

### Atomic displacement parameters (Å^2^)

**Table:**

	*U*^11^	*U*^22^	*U*^33^	*U*^12^	*U*^13^	*U*^23^
Cu1	0.01369 (14)	0.02604 (17)	0.02712 (17)	−0.00543 (12)	0.00426 (12)	−0.00550 (13)
Cu2	0.02511 (17)	0.03088 (19)	0.02237 (16)	−0.00373 (14)	0.00162 (13)	−0.00551 (14)
Cu3	0.01595 (15)	0.0409 (2)	0.0351 (2)	−0.00491 (14)	0.00700 (13)	−0.01941 (16)
Cu4	0.01913 (15)	0.02818 (18)	0.02352 (16)	−0.00774 (13)	0.00518 (12)	−0.00593 (13)
Cl1	0.0216 (3)	0.0303 (4)	0.0323 (3)	−0.0077 (3)	−0.0032 (3)	0.0011 (3)
Cl2	0.0254 (3)	0.0527 (5)	0.0265 (3)	−0.0088 (3)	0.0084 (3)	−0.0048 (3)
Cl3	0.0178 (3)	0.0377 (4)	0.0364 (4)	−0.0069 (3)	0.0055 (3)	−0.0146 (3)
Cl4	0.0320 (3)	0.0325 (4)	0.0270 (3)	−0.0100 (3)	−0.0035 (3)	−0.0038 (3)
Cl5	0.0238 (3)	0.0510 (5)	0.0351 (4)	−0.0049 (3)	0.0145 (3)	−0.0015 (3)
Cl6	0.0554 (6)	0.0713 (7)	0.0395 (5)	0.0348 (5)	0.0163 (4)	0.0098 (4)
N11	0.0136 (9)	0.0250 (12)	0.0317 (12)	−0.0037 (8)	0.0049 (9)	−0.0061 (9)
N12	0.0161 (10)	0.0266 (13)	0.0485 (16)	−0.0053 (9)	0.0071 (10)	−0.0078 (11)
N13	0.0175 (13)	0.049 (2)	0.127 (4)	0.0027 (13)	−0.0002 (17)	0.006 (2)
N21	0.0349 (13)	0.0325 (14)	0.0240 (12)	−0.0034 (11)	−0.0015 (10)	−0.0082 (10)
N22	0.0273 (12)	0.0421 (15)	0.0240 (12)	−0.0001 (11)	0.0020 (10)	−0.0039 (11)
N23	0.0530 (19)	0.051 (2)	0.0406 (17)	0.0300 (16)	−0.0228 (15)	−0.0193 (15)
N31	0.0183 (11)	0.0410 (15)	0.0358 (14)	−0.0054 (10)	0.0040 (10)	−0.0198 (12)
N32	0.0215 (11)	0.0276 (12)	0.0237 (11)	0.0048 (9)	0.0007 (9)	−0.0023 (9)
N33	0.0396 (16)	0.055 (2)	0.057 (2)	0.0174 (15)	−0.0098 (14)	−0.0336 (16)
N41	0.0189 (10)	0.0317 (13)	0.0259 (12)	−0.0082 (9)	0.0033 (9)	−0.0044 (10)
N42	0.0404 (15)	0.0300 (14)	0.0340 (14)	−0.0092 (11)	−0.0094 (12)	−0.0012 (11)
N43	0.090 (3)	0.0332 (17)	0.050 (2)	0.0034 (17)	−0.0176 (19)	−0.0105 (15)
O1	0.0163 (8)	0.0279 (10)	0.0227 (9)	−0.0040 (7)	0.0036 (7)	−0.0080 (8)
O11	0.085 (3)	0.148 (4)	0.061 (2)	−0.048 (3)	0.031 (2)	−0.016 (2)
O12	0.0294 (16)	0.068 (2)	0.273 (7)	0.0062 (16)	−0.022 (3)	0.061 (3)
O13	0.0176 (12)	0.066 (2)	0.161 (4)	−0.0098 (13)	−0.0059 (17)	0.005 (2)
O21	0.0401 (18)	0.073 (3)	0.168 (4)	−0.0018 (17)	−0.004 (2)	−0.021 (3)
O22	0.097 (3)	0.0370 (16)	0.070 (2)	0.0127 (16)	−0.0530 (19)	−0.0170 (14)
O23	0.0467 (16)	0.104 (3)	0.0293 (13)	0.0229 (16)	−0.0096 (11)	−0.0139 (15)
O32	0.0461 (16)	0.088 (2)	0.077 (2)	0.0221 (16)	−0.0221 (15)	−0.0596 (19)
O33	0.0407 (15)	0.080 (2)	0.082 (2)	0.0242 (14)	−0.0056 (14)	−0.0527 (18)
O41	0.055 (2)	0.070 (2)	0.134 (4)	−0.0152 (17)	−0.038 (2)	0.037 (2)
O42	0.108 (3)	0.0457 (17)	0.0396 (15)	−0.0042 (17)	0.0097 (16)	−0.0170 (13)
O43	0.290 (7)	0.045 (2)	0.071 (3)	0.046 (3)	−0.061 (4)	−0.0152 (19)
C11	0.0193 (12)	0.0275 (14)	0.0320 (14)	−0.0053 (10)	0.0064 (10)	−0.0070 (11)
C12	0.0186 (13)	0.0289 (15)	0.053 (2)	0.0019 (11)	0.0050 (13)	−0.0024 (14)
C13	0.0141 (12)	0.0352 (17)	0.065 (2)	−0.0015 (11)	0.0042 (13)	−0.0069 (16)
C14	0.0221 (14)	0.0362 (18)	0.063 (2)	−0.0153 (13)	0.0074 (14)	−0.0067 (16)
C15	0.037 (2)	0.071 (3)	0.084 (3)	−0.022 (2)	0.026 (2)	−0.004 (2)
C16	0.0244 (14)	0.0344 (17)	0.052 (2)	−0.0068 (12)	0.0012 (13)	0.0044 (15)
C21	0.0313 (15)	0.0392 (17)	0.0229 (14)	−0.0076 (13)	0.0035 (11)	−0.0058 (12)
C22	0.0420 (18)	0.0303 (16)	0.0394 (18)	0.0068 (14)	−0.0137 (14)	−0.0113 (14)
C23	0.0357 (16)	0.0406 (18)	0.0284 (15)	0.0114 (14)	−0.0086 (12)	−0.0134 (13)
C24	0.0319 (16)	0.051 (2)	0.0359 (17)	−0.0074 (15)	−0.0003 (13)	0.0097 (15)
C25	0.042 (2)	0.043 (2)	0.069 (3)	0.0006 (17)	0.0065 (19)	0.0059 (19)
C26	0.069 (3)	0.048 (2)	0.0355 (19)	−0.025 (2)	−0.0065 (18)	−0.0030 (16)
C31	0.0176 (12)	0.0325 (15)	0.0245 (13)	−0.0023 (10)	0.0040 (10)	−0.0050 (11)
C32	0.0244 (13)	0.0346 (16)	0.0305 (15)	−0.0031 (12)	0.0001 (11)	−0.0095 (12)
C33	0.0273 (14)	0.0327 (16)	0.0310 (15)	0.0078 (12)	−0.0053 (11)	−0.0106 (12)
C34	0.017 (3)	0.035 (5)	0.030 (5)	0.010 (3)	0.003 (3)	0.000 (3)
C35	0.027 (3)	0.043 (5)	0.035 (4)	0.001 (3)	0.013 (3)	0.003 (3)
O31	0.033 (3)	0.037 (4)	0.052 (4)	0.004 (3)	0.013 (3)	0.011 (3)
C34A	0.022 (4)	0.045 (7)	0.040 (7)	0.019 (4)	−0.010 (4)	−0.005 (5)
C35A	0.023 (4)	0.091 (11)	0.045 (5)	−0.001 (7)	0.010 (4)	0.014 (8)
O31A	0.055 (6)	0.065 (7)	0.041 (4)	−0.023 (5)	−0.007 (3)	0.020 (4)
C36	0.0197 (13)	0.0441 (19)	0.0427 (18)	−0.0038 (12)	−0.0005 (12)	−0.0198 (15)
C41	0.0229 (13)	0.0331 (16)	0.0294 (14)	−0.0129 (11)	−0.0008 (11)	−0.0043 (12)
C42	0.0250 (13)	0.0332 (16)	0.0265 (14)	−0.0074 (11)	0.0038 (11)	−0.0078 (12)
C43	0.0394 (17)	0.0300 (16)	0.0348 (16)	−0.0058 (13)	−0.0052 (13)	−0.0074 (13)
C44	0.059 (2)	0.039 (2)	0.044 (2)	−0.0141 (17)	−0.0106 (18)	0.0024 (16)
C45	0.060 (3)	0.058 (3)	0.060 (3)	−0.004 (2)	−0.009 (2)	0.011 (2)
C46	0.049 (2)	0.0420 (19)	0.0265 (15)	−0.0144 (16)	0.0005 (14)	−0.0057 (14)

### Geometric parameters (Å, º)

**Table:**

Cu1—O1	1.8960 (18)	N31—C31	1.337 (3)
Cu1—N11	1.949 (2)	N31—C32	1.368 (4)
Cu1—Cl1	2.4152 (9)	N32—C31	1.348 (4)
Cu1—Cl3	2.4351 (8)	N32—C33	1.381 (4)
Cu1—Cl2	2.4435 (9)	N32—C34	1.472 (9)
Cu2—O1	1.908 (2)	N32—C34A	1.516 (10)
Cu2—N21	1.955 (3)	N33—O33	1.226 (4)
Cu2—Cl6	2.3579 (10)	N33—O32	1.240 (4)
Cu2—Cl2	2.3726 (9)	N33—C33	1.423 (4)
Cu2—Cl4	2.4351 (9)	N41—C41	1.343 (4)
Cu3—O1	1.9022 (19)	N41—C42	1.368 (4)
Cu3—N31	1.955 (2)	N42—C41	1.342 (4)
Cu3—Cl5	2.3877 (10)	N42—C43	1.390 (4)
Cu3—Cl6	2.4113 (11)	N42—C44	1.484 (5)
Cu3—Cl3	2.4312 (8)	N43—O42	1.207 (4)
Cu4—O1	1.913 (2)	N43—O43	1.209 (5)
Cu4—N41	1.972 (3)	N43—C43	1.426 (5)
Cu4—Cl5	2.4186 (8)	O11—C15	1.404 (6)
Cu4—Cl4	2.4314 (9)	O21—C25	1.370 (5)
Cu4—Cl1	2.4332 (8)	O41—C45	1.458 (6)
N11—C11	1.335 (4)	C11—C16	1.480 (4)
N11—C12	1.356 (4)	C12—C13	1.359 (4)
N12—C11	1.350 (4)	C14—C15	1.501 (6)
N12—C13	1.373 (4)	C21—C26	1.482 (5)
N12—C14	1.481 (4)	C22—C23	1.352 (5)
N13—O12	1.195 (5)	C24—C25	1.510 (5)
N13—O13	1.217 (4)	C31—C36	1.480 (4)
N13—C13	1.426 (4)	C32—C33	1.348 (4)
N21—C21	1.334 (4)	C34—C35	1.490 (10)
N21—C22	1.359 (4)	C35—O31	1.411 (9)
N22—C21	1.356 (4)	C34A—C35A	1.495 (13)
N22—C23	1.376 (4)	C35A—O31A	1.413 (13)
N22—C24	1.469 (4)	C41—C46	1.480 (4)
N23—O23	1.236 (5)	C42—C43	1.343 (5)
N23—O22	1.246 (5)	C44—C45	1.474 (6)
N23—C23	1.425 (4)		
			
O1—Cu1—N11	173.12 (10)	C31—N31—C32	107.4 (2)
O1—Cu1—Cl1	86.13 (6)	C31—N31—Cu3	125.4 (2)
N11—Cu1—Cl1	99.55 (7)	C32—N31—Cu3	127.1 (2)
O1—Cu1—Cl3	84.63 (6)	C31—N32—C33	106.1 (2)
N11—Cu1—Cl3	96.61 (7)	C31—N32—C34	123.8 (6)
Cl1—Cu1—Cl3	112.86 (3)	C33—N32—C34	129.5 (6)
O1—Cu1—Cl2	83.33 (6)	C31—N32—C34A	122.8 (7)
N11—Cu1—Cl2	91.01 (7)	C33—N32—C34A	129.4 (7)
Cl1—Cu1—Cl2	110.37 (3)	O33—N33—O32	124.6 (3)
Cl3—Cu1—Cl2	134.02 (3)	O33—N33—C33	119.6 (3)
O1—Cu2—N21	176.91 (10)	O32—N33—C33	115.9 (3)
O1—Cu2—Cl6	86.06 (6)	C41—N41—C42	107.0 (3)
N21—Cu2—Cl6	91.20 (8)	C41—N41—Cu4	129.2 (2)
O1—Cu2—Cl2	85.06 (6)	C42—N41—Cu4	123.4 (2)
N21—Cu2—Cl2	95.77 (8)	C41—N42—C43	106.5 (3)
Cl6—Cu2—Cl2	132.47 (4)	C41—N42—C44	125.2 (3)
O1—Cu2—Cl4	83.85 (6)	C43—N42—C44	128.2 (3)
N21—Cu2—Cl4	98.64 (8)	O42—N43—O43	124.0 (4)
Cl6—Cu2—Cl4	115.31 (4)	O42—N43—C43	118.0 (3)
Cl2—Cu2—Cl4	109.97 (3)	O43—N43—C43	117.9 (4)
O1—Cu3—N31	176.21 (10)	Cu1—O1—Cu3	110.80 (9)
O1—Cu3—Cl5	85.31 (6)	Cu1—O1—Cu2	108.46 (9)
N31—Cu3—Cl5	96.51 (9)	Cu3—O1—Cu2	108.36 (10)
O1—Cu3—Cl6	84.68 (6)	Cu1—O1—Cu4	108.74 (10)
N31—Cu3—Cl6	91.57 (9)	Cu3—O1—Cu4	109.55 (9)
Cl5—Cu3—Cl6	126.01 (4)	Cu2—O1—Cu4	110.93 (9)
O1—Cu3—Cl3	84.61 (6)	N11—C11—N12	110.5 (3)
N31—Cu3—Cl3	97.52 (7)	N11—C11—C16	125.5 (3)
Cl5—Cu3—Cl3	116.05 (3)	N12—C11—C16	124.0 (3)
Cl6—Cu3—Cl3	115.56 (4)	N11—C12—C13	107.8 (3)
O1—Cu4—N41	175.50 (9)	C12—C13—N12	108.4 (3)
O1—Cu4—Cl5	84.21 (6)	C12—C13—N13	126.4 (3)
N41—Cu4—Cl5	100.26 (7)	N12—C13—N13	125.2 (3)
O1—Cu4—Cl4	83.84 (6)	N12—C14—C15	112.4 (3)
N41—Cu4—Cl4	93.78 (7)	O11—C15—C14	109.9 (3)
Cl5—Cu4—Cl4	113.26 (3)	N21—C21—N22	110.5 (3)
O1—Cu4—Cl1	85.25 (6)	N21—C21—C26	125.7 (3)
N41—Cu4—Cl1	93.57 (7)	N22—C21—C26	123.7 (3)
Cl5—Cu4—Cl1	111.98 (3)	C23—C22—N21	108.1 (3)
Cl4—Cu4—Cl1	131.90 (3)	C22—C23—N22	108.5 (3)
Cu1—Cl1—Cu4	79.38 (2)	C22—C23—N23	125.7 (3)
Cu2—Cl2—Cu1	79.70 (2)	N22—C23—N23	125.6 (3)
Cu3—Cl3—Cu1	79.95 (3)	N22—C24—C25	111.6 (3)
Cu4—Cl4—Cu2	80.61 (2)	O21—C25—C24	111.5 (4)
Cu3—Cl5—Cu4	80.86 (3)	N31—C31—N32	110.3 (2)
Cu2—Cl6—Cu3	80.74 (3)	N31—C31—C36	125.6 (3)
C11—N11—C12	107.5 (2)	N32—C31—C36	124.2 (2)
C11—N11—Cu1	123.7 (2)	C33—C32—N31	107.8 (3)
C12—N11—Cu1	128.4 (2)	C32—C33—N32	108.4 (3)
C11—N12—C13	105.8 (2)	C32—C33—N33	126.4 (3)
C11—N12—C14	124.5 (3)	N32—C33—N33	125.1 (3)
C13—N12—C14	129.7 (3)	N32—C34—C35	110.2 (7)
O12—N13—O13	122.9 (3)	O31—C35—C34	110.9 (8)
O12—N13—C13	117.5 (3)	C35A—C34A—N32	112.3 (8)
O13—N13—C13	119.7 (4)	O31A—C35A—C34A	111.8 (9)
C21—N21—C22	107.3 (3)	N42—C41—N41	110.2 (3)
C21—N21—Cu2	126.3 (2)	N42—C41—C46	124.1 (3)
C22—N21—Cu2	126.1 (2)	N41—C41—C46	125.7 (3)
C21—N22—C23	105.6 (3)	C43—C42—N41	108.6 (3)
C21—N22—C24	125.2 (3)	C42—C43—N42	107.6 (3)
C23—N22—C24	127.8 (3)	C42—C43—N43	124.9 (3)
O23—N23—O22	125.4 (3)	N42—C43—N43	127.3 (3)
O23—N23—C23	118.8 (4)	C45—C44—N42	110.4 (3)
O22—N23—C23	115.9 (4)	O41—C45—C44	109.5 (4)

### Hydrogen-bond geometry (Å, º)

**Table:**

*D*—H···*A*	*D*—H	H···*A*	*D*···*A*	*D*—H···*A*
O41—H41*A*···O31^i^	0.89 (2)	2.13 (3)	2.738 (8)	125 (2)

Symmetry code: (i) *x*+1/2, *y*−1/2, *z*.
